# Alterations of Signaling Pathways Related to the Immune System in Breast Cancer: New Perspectives in Patient Management

**DOI:** 10.3390/ijms19092733

**Published:** 2018-09-12

**Authors:** Andrea Nicolini, Paola Ferrari, Lucrezia Diodati, Angelo Carpi

**Affiliations:** 1 Department of Oncology, Transplantations and New Technologies in Medicine, University of Pisa, 56126 Pisa, Italy; paolaferrari2266@libero.it (P.F.); lucreziadio@live.it (L.D.); 2 Department of Clinical and Experimental Medicine, University of Pisa, 56126 Pisa, Italy; angelo.carpi@med.unipi.it

**Keywords:** breast cancer, immune response, signaling pathways, tumor initiating cells, cancer stem cells, TNF, NF-κB, CD47 upregulation, STAT-1, STAT-3

## Abstract

In recent years, immune manipulation for cancer treatment, including breast cancer, has been increasingly gaining consent, and many attempts have been made, mainly by either strengthening the immune response (IR) or by inhibiting immune evasion. Therefore, elucidating the related mechanisms is of importance due to the potential to improve the management of cancer patients by immunotherapy. This review article summarized some recent experimental studies, which have discovered novel alterations of signaling pathways related to the immune system in breast cancer. These altered signaling pathways have been grouped according to the general biological mechanism involved: tumor-initiating cells (TICs), cancer stem cells (CSCs), immune evasion, tumor growth and progression, prediction of clinical outcome and prediction of response, or resistance to chemotherapy. These altered pathways related to the immune system open clinical opportunities for the prognosis or treatment of patients. Many of these pathways are related to the origin of breast cancer and immune evasion. We recommended development of new drugs which act on these molecular pathways, and the designing of clinical trials to be carried out mainly in breast cancer patients who required adjuvant treatment.

## 1. Introduction

The immune response (IR) to cancer by the immune system, can be innate or adaptive. The innate immune response, which is mostly engaged upon the development of cancer, is sustained by natural killer (NK) cells, natural killer T (NKT) cells, dendritic cells (DCs), macrophages, neutrophils, basophils, eosinophils, mast cells, human intraepithelial lymphocytes (IELs), and gamma delta T cells. Adaptive IR is sustained by B lymphocytes, CD4+ helper T lymphocytes, and CD8+ cytotoxic T lymphocytes (CTLs). These differ from innate leucocytes because unlike germ-line encoded receptors, they express somatically generated antigen-specific receptors following random gene rearrangement [1]. Plasma cells are effector B lymphocytes following T helper activation. Adaptive immunity allows a wider and more flexible repertoire of responses than innate immunity does, and plays a major role during the pathological progression of cancer. Tumor growth and diffusion is sustained by many altered molecular pathways related to various biological capabilities. These alterations are commonly due to crosstalk among different cellular components within the tumor environment, including immune cells, and are often mediated by different cytokines, mainly interferons (INFs), interleukins (ILs), chemokines and growth factors [2]. Alterations of relevant signaling pathways involving cells and/or immune response mediators and immune signatures, have recently been reported. These observations contribute to a better understanding of the origin of breast cancer, immune evasion, progression, prognosis, and the prediction of response to therapy. We review these studies with the principal aim of describing new possible targets for prognosis and/or treatment.

## 2. Role of Myeloid Derived Suppressor Cells (MDSCs) on Tumor Initiating Cells (TICs) and of INFs on Cancer Stem Cells (CSCs)

Tumor-initiating cells (TICs) and cancer stem cells (CSCs) have properties similar to those observed in normal adult tissue, and represent the basis of the “cancer stem cell” theory of breast cancer origin [3,4]. 

### 2.1. MDSCs and TICs-Notch Signaling

In an experimental study [5], it was shown that oncogenic signaling linked to TICs governs the capability to attract and accumulate MDSCs. There is wide heterogeneity regarding the additional presence of MDSCs in the same type of malignancy. Granulocyte colony stimulating factor (G-CSF) [6], granulocyte-macrophage colony-stimulating factor (GM-CSF) [7], IL-1β [8], IL-6 [9], prostaglandin 2 (PG2) [10], INF-γ [11], IL-4 [12], and vascular endothelial growth factor (VEGF) [13] are the cytokines and factors, which are most often involved in the accumulation of MDSCs. However, the precise mechanisms governing the propensity of individual tumors to attract MDSCs are largely unknown. Experiments have been conducted in a variety of syngeneic mammary tumors generated, either by spontaneous tumorigenesis (MMTV-WNT1, WNT1-iFGFR, and P53-PTEN double-knockout (DKO)), orthotopic transplantation of primary tumor tissues (P53N series), or cell suspensions (67NR, 4TO7 and 4T1). The accumulation of CD11b+ Gr1+ cells was systemic, and concentrations in peripheral blood were closely correlated with tumor-infiltrating myeloid cells. While the intermodel variation of CD11b+ Gr1+ cells ranged from 50 to 100-fold, the variation ranged from 2 to 5-fold within individual models. This indicated a stable behavior for each tumor line. In particular, MDSC accumulation was correlated with the Akt/protein kinase B (AKT) mammalian target of rapamycin (mTOR) and the mitogen-activated protein kinase (MAPK) pathways across the different models, although not with the P53 series, which was correlated only with the AKT-mTOR pathway. This suggests that the mTOR pathway is a driver of MDSC accumulation. An increased expression of G-CSF was also observed. The fact that G-CSF is a downstream target of the mTOR pathway in tumor cells, suggests that it is a mediator of mTOR-driven MDSC accumulation. Additionally, genomic analyses support the role of G-CSF and MDSCs in mTOR-driven immune suppression and tumor progression. Findings from The Cancer Genome Atlas (TCGA) [14] suggest that human tumors with an activated mTOR pathway have a higher infiltration of MDSCs, a decreased infiltration of T cells and a worse prognosis. Furthermore, cancer cells which overexpress G-CSF and have increased activity of the m-TOR pathway are enriched in TICs, and TICs exhibit enhanced G-CSF production, providing evidence for the importance of the connection between TICs, the mTOR pathway, G-CSF, and MDSCs. Finally, the authors reported that (a) the G-CSF expression was correlated with Notch target genes in the TCGA dataset; (b) MDSC co-culturing significantly increased the expression of multiple Notch stemness-related genes in a contact-dependent fashion; (c) transient inhibition of mTOR signaling decreased TIC frequency, partially mediated by MDSCs. They concluded that MDSCs enrich TICs via Notch signaling in cancer cells.

### 2.2. INF, TIC Activities, and CSCs

**Figure 1:**
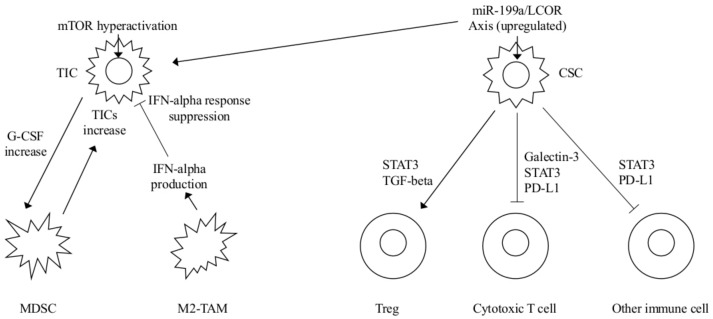
Main interactions of TICs/CSCs with immune cells and their mediators. CSCs recruit anti-inflammatory M2 tumor-associated macrophages (TAMs) which suppress adaptive immune responses. Besides, CSCs induce Treg expansion and effector fraction through the production of TGF-beta and STAT3 concomitant with cytotoxic Tcell apoptosis through galectin-3 production. A direct cell-to-cell contact between CSCs and cells of the immune system mediated by cell surface expression of PD-L1 also suppresses immune cell function (also see text).

A previous study [15] identified the crucial role of microRNA (miR)-199a in promoting mammary stem cell (MaSC) and TIC properties, by direct repression of the ligand-dependent nuclear receptor co-repressor (LCOR). The miR-199a–LCOR axis has been found to be a conserved molecular pathway in normal and cancerous stem cells. The authors also showed that miR-199a functionally promotes MaSC. The MaSC-enriched basal population (denoted as P4) showed a higher expression of miR-199a, than the luminal population (denoted as P5) of primary mammary epithelial cells (MEC). This finding was confirmed by in situ hybridization (ISH) in the mammary gland. Additionally, miR-199a-induced stem cell-like gene signatures were upregulated in all of the evaluated TIC/CSC populations. It was then shown that LCOR inhibits MaSC activity, is a direct target of miR-199a, and is downregulated in MaSCs and CSCs. Other findings suggest that the miR-199a–LCOR axis promotes TIC activity in ER-negative breast cancer, and that miR-199a–LCOR modulates the INF-α response of normal stem cells and CSCs. When using mammosphere assays, a differential response of P4 and P5 cells to INF-α occurred in vivo. In particular, stem cell-related genes were upregulated in P4 spheres and downregulated in P5 spheres, while luminal differentiation genes were upregulated in P5, but not in P4, upon INF-α treatment. These and other findings showed that the miR-199a–LCOR axis modulates the sensitivity of normal and cancerous stem cells to the differentiation and senescence effects of INF-α. In different types of breast tumors, high levels of INF-α and increased infiltration of INF-α positive macrophages were observed. The conditioned media (CM) of mammary gland macrophages significantly increased P4, and decreased P5, sphere formation, but this effect was lost following miR-199a overexpression in P5 cells. The effects of CM from mammary gland macrophages on P5 spheres were lost following treatment with antibodies, which neutralize against α/β-INFs, demonstrating that these effects were mediated by INFs. The authors conclude that both normal and cancerous stem cells are protected from immune or autocrine/paracrine INF-mediated suppressive effects, through the overexpression of miR-199a and the decrease of LCOR levels. We have recently proposed a refined patho-biological model of cancer, considering tumor growth and immune evasion as closely related and promising potential targets for a new therapeutic strategy [16]. This new strategy is based on the hypothesis that larger tumor burdens correspond to higher immune system inhibition. Therefore, drugs contrasting immune evasion should be tested in conditions with low tumor burden, such as adjuvant settings. TICs and CSCs play a major role in the origin of cancer and likely also in relapses, because they both correspond to highly proliferative cellular phenotypes. An increased treatment efficacy could be experimentally evaluated by contrasting TICs and/or CSCs. The results reported in a study of TICs [5] showed that the inhibition of the phosphatidyl-inositol-3 kinase (PI3K)-mTOR pathway may help to overcome immune suppression and increase the efficacy of other immunotherapies. A recent study using a PI3K inhibitor showed synergistic effects with an immune checkpoint blockade [17]. The association of conventional adjuvant PI3K-mTOR pathway inhibitors, with drugs blocking fibroblast growth factor receptor (FGFR) or G-CSF, should inhibit the accumulation of MDSC with a substantial concomitant decrease in TICs. The addition of checkpoint inhibitors, which induce immune tolerance by a different mechanism, is another experimental option. Findings reported on CSCs and INFs [15], suggest that a low INF response likely provides a benefit to tumors in different types of cancer. Increased treatment efficacy should be attained by conventional adjuvant treatments, combined with drugs developed for inhibiting the miR-199a–LCOR axis. The inhibition of the miR-199a–LCOR axis is expected to favor CSCs differentiation. Combinations of these drugs should be tested in animal models with aggressive breast cancer phenotypes, to investigate their capacity to inhibit or delay the occurrence of metastases. Consistent with our proposed model, the associations between these drugs could be validated in high-risk breast cancer patients in an adjuvant setting. A summary of the main interactions between TICs and CSCs with immune cells and their mediators, are shown in Figure 1.

### 2.3. Stabilization of PD-L1, Up-Regulation of CD47 in Cancer Cells, and ShcA Signaling as Mechanisms of Immune Evasion

#### 2.3.1. PD-L1 Stabilization

While programmed cell death protein-1 (PD1) is mainly expressed in immune cells [18], programmed death-ligand 1 (PD-L1), also known as B7 homologue 1 (B7 H1), is a trans-membrane protein in cancer cells and macrophages, which plays a principal role in inducing immune tolerance [19] by binding to the PD1 receptor. N-glycosylation of membrane receptor proteins can affect protein structure and function, thus becoming crucial for protein–protein interactions, such as those between ligands and receptors. Previous findings [20] showed that PD-L1 is at least partly glycosylated in cancer cells through the PD-L1 extracellular domain, and that the glycosylation of N192, N200, and N219 contributes to PD-L1 protein stability. Glycogen synthase kinase 3β (GSK3β) is a serine/threonine kinase, which through direct phosphorylation, facilitates the recognition and degradation of non-glycosylated PD-L1 by β-Trcp ubiquitin ligase in vitro and in vivo. Glycosylation antagonizes GSK3β binding. It has been found that PD-L1 had a consensus GSK3β phosphorylation motif adjacent to N192, N200, and N219, which was critical for PDL1 stability. The reported findings together suggest, that the region containing N192, N200, and N219 residues is necessary for the binding of PD-L1 to GSK3β, and that glycosylation of the same residues antagonizes PD-L1 and GSK3β interaction. Other findings, in an in vivo mouse model, support the hypothesis that the stabilization of PD-L1 by the inactivation of GSK3β increases tumor immunosuppressive function, promoting tumor cell survival. Among the growth factors known to inhibit GSK3β activity, only the epidermal growth factor (EGF) and a few other epidermal growth factor receptor (EGFR) ligands, induced PD-L1 expression. This induction mainly occurred at a post-translational level, since EGF did not affect PD-L1 mRNA expression. EGF stabilized PD-L1 via the inactivation of GSK3β in basal-like breast cancer. The inhibition of EGF signaling by gefitinib, destabilized PD-L1 and enhanced antitumor T cell immunity. The authors conclude that ubiquitination and glycosylation are pathways closely linked with the regulation of PD-L1. 

#### 2.3.2. CD47 Upregulation

Tumor cells that express CD47, a cell surface glycoprotein, inhibit phagocytosis by binding to its receptor, signal-regulatory protein (SIRP) alpha, situated on macrophages and other immune cells [21]. In all types of cancer [22], CD47 transcript and protein expression is abnormally upregulated, thus protecting the cancer cells from being recognized and eliminated by macrophages [23]. Cis-regulatory regions and genomic enhancers are often described as “switches,” capable of regulating gene transcription [24]. Recently, super-enhancers (SEs), which commonly consist of long stretches of DNA containing clusters of enhancers, known as constituent enhancers, have been discovered [25]. It has been reported that SEs play a key role in upregulating master regulatory genes in different diseases, including cancer [26]. In many cancers, SEs are acquired by critical genes that control and define tumor cell identity [25,26]. Betancur et al. were the first to analyze the CD47 regulatory genomic landscape, to locate CD47 distal cis-regulatory regions (distal enhancers or SEs) and their upstream activators. SEs have been associated with CD47 in breast and other cancers, and constituent enhancers were identified. It was observed that signal transducers, activators of transcription (STAT) 3, 5, and 6, nuclear factor kappa B (NF-κB) and/or PPAR transcription factors could play a key role in the activation of CD47 expression by binding to the CD47 E5 constituent enhancer. However, following shRNA transduction, the expression of the CD47 transcript was significantly decreased by NF-κB1 and a deletion, including an NF-κB site inhibited E5 activity. Moreover, the blockade of tumor necrosis factor (TNF)-α reduced CD47 and increased phagocytosis, suggesting that the TNF-NF-κB signaling pathway directly governs CD47, by interacting with a constituent enhancer placed within a CD47-associated SE specific to breast cancer. This study elucidated another mechanism by which breast cancer escapes immune surveillance, which involves the upregulation of CD47 by SE, which inhibits phagocytosis signaling.

#### 2.3.3. Type III Chaperone Protein ShcA (ShcA) Signaling

The main aim of Reference [27] was to investigate the role of Shc1 (or ShcA) function, in governing the balance between STAT1 and STAT3 activities. Shc1 is a scaffold protein, which functions as a central regulator of tyrosine kinases. It has been shown that tyrosine kinases increase immune suppression [28], and that their inhibition can reactivate antitumor IRs [29,30]. Tyrosine kinases transduce oncogenic signals via signaling intermediates, and require downstream ShcA signaling to evade antitumor immunity [31]. The study elucidated the mechanism by which ShcA transduces immunosuppressive signals. In particular, it was shown that ShcA phosphotyrosine motifs augment immune suppression by limiting STAT1-driven antitumor immunity, whilst simultaneously increasing STAT3 immunosuppressive signals. It was shown that impaired Y239/Y240-ShcA phosphorylation selectively diminished STAT3 activation in breast tumors, sensitizing them to immune checkpoint inhibitors and tumor vaccines. The authors concluded that the inhibition of pY239/Y240-Shc-A dependent STAT3 signaling, may be an effective technique to sensitize breast cancer to different immunotherapies. However, inherent functional redundancy within the tyrosine kinome promotes resistance and is the main limit to the efficacy of tyrosine kinase inhibitors [32]. The reported relationships between IR and TICs, CSCs, and immune evasion, are shown in Table 1. The first report on immune evasion [20] showed a novel interchange between glycosilation and phosphorylation, which regulates ubiquitination and PD-L1 degradation. This regulatory mechanism appears to be crucial for basal-like breast cancer (BLBC) cells to escape immune surveillance via PD-L1/PD-1 interaction. The authors suggested that targeting PD-L1 stabilization could be a novel strategy for contrasting BLBC-mediated immunosuppression, and that it may potentially apply to other cancer types. In another study [21], CD47 expression in breast cancer cells was shown to be enhanced by the TNF-NF-κB1 inflammatory pathway, and its inhibition by blocking antibodies increased the recruitment of the innate immune system and phagocytosis. The authors concluded that it is critical to understand the factors regulating CD47 gene expression at the transcriptional level, since this could lead to alternative targeted cancer gene therapies, allowing for the recruitment of the host’s innate immune system, to specifically recognize and destroy cancer tumorigenic cells. In the aforementioned study [27], changes in specific signal transduction pathways downstream of Y239/Y240-ShcA phosphorylation were found to affect sensitivity to immune therapies in breast cancer. Thus, preventing Y239/Y240-ShcA phosphorylation by specific inhibitors or by competitive binding, may sensitize breast tumors to immunotherapies. Targeting PD-L1 degradation, inhibiting CD47 expression, and preventing Y239/Y240 ShcA phosphorylation by using specific drugs, should allow tumor immune suppression to be contrasted differently from PD1-PD-L1 inhibitors. These should be tested using the same experimental models used to date (for metastatic melanoma or lung cancer), to verify a possible synergizing activity. Additionally, according to our proposed model, these should be tested in a breast cancer adjuvant setting together with conventional therapies.

### 2.4. Altered Intra-and Inter-Cellular Signaling in the Immune Microenvironment Affects Tumor Growth and Progression

#### 2.4.1. C-C motif Chemokine Receptor (CCR)7 and Its Chemokine Ligands (CCL)19/(CCL)21

A cohort of 866 patients with unselected primary operable invasive breast cancer (IBC), with detailed clinic-pathological profiles, were investigated using an immunostaining procedure, as described in Reference [33]. The principal aim was to better understand the previous findings regarding CCR7 and its ligands, the lymphoid homing CCL19/CCL21. In several studies, CCR7 expression by IBC cells has been found to induce tumor growth, proliferation and metastasis, as well as angiogenesis and lymphangiogenesis [34,35] in animal models [36]. Moreover, mRNA and the protein expression of CCR7 were most often linked to tumors associated with the lymph nodes [37,38,39]. However, the METABRIC analysis found that this association was limited to the luminal B molecular subtype [40]. In malignant cells, CCR7 staining occurred as membranous and cytoplasmic staining that was scored independently. A significant association occurred in cases with more than three positive nodes when CCR7 was located in a singular compartment, membrane, or cytoplasm. Subsequently, using the St. Gallen recommendations for defining molecular sub-types, and after considering HER2 positive cases in a separate group, significant (*p* = 0.009) enrichment in higher staining for cytoplasmic CCR7 was found in HER2+, compared to all others. Moreover, both HER2 positivity and high grade were found to be positively correlated with axillary lymph node involvement (*p* = 0.03 and *p* < 0.001, respectively). The authors suggested that the strong association between CCR7 and lymph node status reported in previous studies, was likely affected by a biased proportion of HER2 positivity or high grade in the lymph node positive cases. The subcellular localization of CCR7 is critical to its function. Membrane-associated CCR7 has been reported to be responsible for an active chemotactic response to macrophages [41] and T lymphocytes. In the study, a positive association was found between high membrane and total CCR7 expression, and the presence of FOXP3+ tumor infiltrating cells in the adjacent stroma, as well as a trend for higher intratumoral FOXP3+ cells. Additionally, a higher number of intratumoral CD68+ cells (macrophages) were observed in tumors with high membrane CCR7 expression. These findings suggested an interaction between tumor membrane CCR7 expression and the recruitment of macrophages and T regulatory lymphocytes within the tumor microenvironment. Conversely, cells with only cytoplasmic CCR7, unlike those with membrane CCR7 localization, did not respond to CCL19/CCL21 stimulation, which may reflect a different role independent of chemotactic interaction [42]. In fact, cytoplasmic and total CCR7 staining were inversely associated with the presence of CD3+ cells in the distant stroma, and the association was more significant in ER- HER2- tumors. The authors concluded that, although the study did not find evidence that the CCR7 protein is a useful predictor of lymph node metastasis, disease-free recurrence, or breast cancer-specific survival, its expression was associated with an altered tumor microenvironment, which might affect the capability of tumors to spread through lymphatic vessels.

#### 2.4.2. Annexin 1 (ANKA1) and Macrophages

The importance of Annexin 1 (ANKA1), a 37-kDa protein belonging to the annexin superfamily, was elucidated in a previous study [43]. This protein is highly expressed in metastatic and triple-negative breast cancer (TNBC), where it is involved in tumor development and progression [44]. In breast cancer, ANXA1 can also be associated with NF-κB, increases c-Myc activity, leading to the inhibition of miR196a transcription and promotes breast cancer migration and metastasis [45,46]. The biological action of ANXA1 is mediated by formyl peptide receptors (FPRs). It has been shown that ANXA1 by FPR2 activation, stimulates breast cancer proliferation [47]. Breast tumors are usually widely infiltrated by T cells, tumor-associated macrophages (TAMs), which are recruited by many different growth factors, and chemokines produced by the cancer cells themselves. In the aforementioned study, CM of 4T1 and 67NR murine breast cancer cells, were shown to be able to polarize macrophages of the M2-phenotype. This phenotype drives angiogenesis, tumor progression, and induces Th2-type adaptive immune suppression, likely due to the secretion of IL-10 and transforming growth factor β (TGF-β) [48]. In the absence of ANXA1, the polarization of macrophages in tumors is skewed to the M1 phenotype. Using a cytokine array, it was observed that 4T1-CM had much higher levels of CCL5 compared with serum-free media, suggesting that CCL5 induces ANXA1 expression and modulates macrophage polarization. It was additionally observed that ANXA1 and 4T1-CM increase extracellular regulated MAP kinase (ERK) and NF-κB activation via FPR2. This indicated that the FPR2-ERK signaling axis is involved downstream of ANXA1.

#### 2.4.3. Inflammatory Cells, NR4A1 TGF-β/SMAD Signaling

The relationships between cancer and sites of infection and chronic inflammation has been widely documented, and there is increasing evidence suggesting the tumor-promoting activity of immune cells, including inflammatory cells [49]. During tumor progression, immune cells (monocytes, neutrophils, Tregs, and MDSCs) are attracted to tumors, and take part in the neoplastic process through the secretion of various cytokines [49,50]. A study [51] showed that in aggressive breast cancer, nuclear orphan receptor NR4A1 expression is strongly induced by the inflammatory cytokines IL-1β and TNF-α. Consistent with this finding, elevated NR4A1 expression was found to occur in breast cancer patients with high immune infiltration. The TGF-β pathway and NR4A1 have been shown to play a key role in maintaining cellular homeostasis, and tumor suppressive and tumor promoting activities have been reported for both of these factors. In the study, NR4A1 was found to act as a strong signaling activator of TGF-β/SMAD by inducing the degradation of SMAD7, which leads to increased SMAD signaling by prolonging levels of the active TGF-β type I receptor (T-βRI) on the plasma membrane. TGF-β signals via specific complexes of type I and type II Ser/Thr kinase receptors. The activated T-βRI induces SMAD2/3 phosphorylation, followed by the accumulation of SMAD4 heterodimers in the nucleus, which regulates the expression of target genes [52,53]. NR4A1 was found to promote TGF-β-induced epithelial-mesenchymal transition (EMT) and cell migration. Additionally, using an immune-histochemical analysis of tissue samples from 175 breast cancer patients, it was found that NR4A1 expression correlated with poor prognosis. In conclusion, this study demonstrated that inflammation-induced NR4A1 strongly promotes TGF-β-mediated breast cancer cell invasion and metastasis. The main findings of this section are shown in Table 2. The findings of the two experimental studies described in the section on tumor growth and metastasis, suggest that FPR2-ERK signaling [43], TGF-β, and NR4A1 [51] are candidate targets for novel treatments of overt metastatic disease. In the former study, the FPR2-ERK signaling induced a M2 phenotype in macrophages, which accumulated at the tumor site, promoting tumor growth and vasculature [54,55], as well as breast cancer proliferation [47]. NR4A1 is a strong activator of TGF-β/SMAD signaling promoting TGF-β mediated EMT, the migration of breast cancer cells and invasion and metastasis in vitro and in vivo. Therefore, targeting FPR2-ERK signaling, anti-TGF-β treatment, and or NR4A1 inhibitors could be novel therapeutic strategies. These novel strategies, which have high anti-proliferative potential, according to our proposed model, should be tested alone as maintenance therapy in breast cancer patients in partial or complete response, following conventional chemotherapy.

### 2.5. Prediction of Clinical Outcome

#### 2.5.1. The NF-κB Pathway

Hereditary breast cancers arise from mutations of the tumor suppressor gene *BRCA1*, and share histological features with triple-negative breast cancer (TNBC) and basal-like breast cancers (BLBCs). BRCA1 function can be suppressed by both mutation and the downregulation of expression (also called “BRCAness”). Tumors related to *BRCA1* mutation often overexpress IR genes [56], and co-occur with large numbers of infiltrating lymphocytes [57]. Moderate to large lymphocytic infiltration has also been reported in about half of all TNBCs [58], and this finding is associated with a good clinical outcome [56,59]. This suggests that the immune system plays a significant role in modulating TNBC responses. The NF-κB pathway mediates the response of cancer cells to external stimuli, and its activation affects cancer survival, invasion, metastasis, and the IR. A study [60], observed NF-κB hyperactivity with an enhancement of NF-κB target genes. This was observed in a subset of *BRCA1*-mutant and TNBCs, concomitant with BRCA1 dysfunction due to an increase in reactive oxygen species (ROS), and was associated with a good outcome. In particular, a BRCA1-deficient, NF-κB-driven immune signal (“NF-κB on”) was identified concomitant with a favorable “M1-type” macrophage tumor microenvironment, promoting active cytotoxic CD8+ infiltration. The authors concluded that the NF-κB-driven signal from within the tumor cells promotes a M1/Th1 microenvironment; these cells then produce their own signals, which enhance and maintain this effect and influence responses to chemotherapy and overall outcome. In the “NF-κB off” cell lines, a more M2-like microenvironment was found to prevail, concomitant with the upregulation of the IGF and AR signaling pathways.

#### 2.5.2. Prognostic HTICS Signature Involving an IR

In another study [61], the pathways and genes that underlie the prognostic capability of a 17-gene HER2-TIC-enriched signature (HTICS) were investigated. Around 20% of breast cancers show amplification of *HER2/ERBB2/NEU*, either with or without the expression of ER-α. Some HER2+ ER-α breast cancer patients do not develop metastases, and in these patients, surgical tumor removal together with local radiation or conventional chemotherapy can be sufficient as frontline therapy. However, other tumors are more aggressive and require well-tailored adjuvant treatment. A validated prognostic signature from primary biopsies of such tumors, can assist clinicians to choose more appropriate adjuvant treatments. In the study, cell proliferation, IR, and cell migration were identified as three critical biological pathways, which determine the prognostic capability of HTICS in HER2+ ER-α breast cancer patients. It was additionally demonstrated that most genes capable of replacing this signature shared the same pathways, as well as a 6-gene minimal core that maintained significant prognostic power, albeit to a lesser degree than HTICS. The expression of HTICS genes and their substitutes, could be reliably assessed in formalin-fixed paraffin-embedded tissues derived from primary tumor biopsies using NanoString technology. Publically available cohorts with gene expression and clinical outcome were used to assess the prognosis. Retrospective analysis of a small cohort of HER2+ ER-α patients treated with trastuzumab showed that HTICS+ subjects had a worse prognosis than HTICS subjects, but benefitted from trastuzumab therapy. The authors note that the presence of an IR pathway, even in the minimal 6-gene core, is particularly interesting given the effect of the immune system on the therapeutic effect of trastuzumab, and that combination therapy with trastuzumab and immune-modulating drugs may therefore be highly synergistic for HTICS+ HER2+ ER-α patients. Conversely, HTICS-HER2+ ER-α patients showed a good prognosis, and may be excluded from receiving anti-HER2 treatment.

#### 2.5.3. Long-Noncoding (Lnc) RNAs

Another experimental study [62], which investigated the molecular mechanisms of tumorigenesis, identified a cluster of oncogenic LncRNAs that were upregulated in breast cancer tissue and associated with worse survival outcomes. These oncogenic LncRNAs are involved in regulating immune system activation (lymphocyte activation, T cell receptor signaling pathway, etc.), and the TGF-β and Jack-STAT signaling pathways. Moreover, three sub-type-specific LncRNAS were found to be associated with HER2, triple-negative, and luminal B molecular subtypes of breast cancer. The mechanisms of the upregulation of these LncRNAs, were mainly related to gene amplification. Furthermore, some of these LncRNAs were positively regulated, and others negatively regulated, by estrogen. However, the detailed mechanisms of these oncogenic LncRNAs were not elucidated. This section is of particular interest, since the risk–benefit ratio is something that needs to be continuously evaluated as candidate approaches move towards clinical application.

### 2.6. Prediction of Response or Resistance to Chemotherapy

#### 2.6.1. Two Immune-Based Gene Modules

Another study [63] investigated the predictive value of gene expression signatures from fine needle aspiration biopsy (FNAB) and core biopsy for chemotherapy, in a cohort of 109 patients enrolled in the TEX trial. This was a multicenter, randomized phase III clinical trial (ClinicalTrials.gov identifier NCT01433614), where epirubicin and paclitaxel were administered, with or without capecitabine, as a first-line treatment for locally advanced inoperable or metastatic breast cancer. Six gene expression signatures were investigated, from which six corresponding module scores, Immune Module 1, Immune Module 2, ESR1 module, Proliferation module, PIC3CA module, and TP53 module, were defined. The objective response to chemotherapy was significantly (*p* = 0.04) associated with an immune module score. Sub-group analysis showed that this association was limited to patients with ER-positive or luminal tumors (*p* = 0.012 and *p* for interaction = 0.04), and gene set enrichment analysis (GSEA) confirmed an enrichment in immune-related gene sets in responders relative to non-responders. Immune Module 1 and Immune Module 2, predicted sensitivity to chemotherapy in ER-positive and luminal breast cancer better than other gene signatures. Based on these findings, the authors concluded that predictive information is dependent on the presence of an activated immune microenvironment, and not on the expression of specific genes, and that the importance of the immune infiltrate was confirmed by an independent and unbiased GSEA, showing that gene sets related to crosstalk between immune cells and cancer cells, PD-1 signaling, and INF-γ signaling, were among the most highly enriched in chemo-sensitive tumors.

#### 2.6.2. Plasma Cells Inhibit Immunogenic Cell Death (ICD)

Despite that oxaliplatin cannot cross the blood–brain barrier to treat brain metastasis [64], it has gained a consolidated role in aggressive TNBCs, advanced settings, and in heavily pretreated breast cancer patients [65,66,67]. Oxaliplatin is a chemotherapeutic medication, which also induces immunogenic cancer cell death (ICD) by promoting cancer-specific IR [68]. An experimental study [69] suggested that immunosuppressive plasma cells inhibit the ICD induced by oxaliplatin. The study was carried out in three different mouse prostate cancer models. All of them were refractory to oxaliplatin, unless genetically or pharmacologically depleted of B cells. The crucial immunosuppressive B cells were plasmocytes expressing IgA, IL-10, and PD-L1, which were recruited through the TGF-β receptor (TGF-βR) signaling. Removing these cells allowed the CTL-dependent eradication of oxaliplatin-treated tumors. The main findings relating to the immune system and the prognosis or prediction of response to therapy, are shown in Table 3. In the first paper of the last section [63], overexpression of immune-related genes, predicted chemosensitivity in patients with advanced breast cancer (ABC) and luminal molecular sub-type. This finding may also have implications for the design of combined chemo-immunotherapies. This last aim is being actively pursued in phase III trials by ABC (ClinicalTrials.gov identifiers NCT02819518 and NCT02425891). In the aforementioned study [69], the elimination or inhibition of tumor-infiltrating IgA+ plasmocytes, seems to be crucial for a successful immunotherapeutic response to oxaliplatin.

## 3. Perspectives and Conclusion

In the last decade, immune manipulation for cancer treatment has attracted great attention and expectation. Enhancing the IR to cancer and inhibiting immune cell suppression have been the main approaches. Specific molecular pathways for these actions have been described. To date, the results of clinical trials of drugs which interfere with some of the reported molecular pathways, mainly refer to CTL4 and the PD 1/PDL1 checkpoints, which account for “exhausting” CTLs or as a “break” to their function. Promising results have been shown with checkpoint inhibitors, mainly in melanoma [70] and lung cancers [71,72]. Their use alone or in combination with other treatments is also under investigation in breast cancer. However, as highlighted here, additional mechanisms involving the immune system have been reported, which could lead to substantial advancement in the field. Novel molecular signaling pathways related to immune system function in breast cancer have been reviewed. Many of these pathways are related to the origin of breast cancer and immune evasion. We recommend developing new drugs, which act on these molecular pathways, and designing clinical trials to be carried out mainly in breast cancer patients who require adjuvant treatment.

## Figures and Tables

**Table 1:** Immune Response (IR) Mechanisms and Tumor Initiating Cells (TICs), Cancer Stem Cells (CSCs) or Immune Evasion.

IR Factor or Mediator	Mechanism	Result	Perspective	Ref.
MDSCs	Hyperactivated Akt-mTOR pathway G-CSF increased expression MDSC mediated Notch stemness-related genes upregulation	TICs mediated MDSCs accumulation Increased TICs frequency	mTOR plus checkpoint inhibitors FGFR or G-CSF inhibitors	[5]
INF-α	miR-199a overexpression LCOR repression and modulation of the INF-α mediated suppressive effects	CSCs protected by INF-mediated effects MaSC-enriched basal vs luminal population	INF-α plus miR-199-LCOR targeting as adjuvant therapy	[15]
PD-L1	N192, N200, N219 glycosylation induces PD-L1 stability and antagonizes PD-L1 GSK3-β interactions as well as EGF and other EGFR ligands	Immunesuppression	Targeting PD-L1 stabilization	[18]
CD47	TNF-NF-κB mediated CD47 upregulation by SEs CD47 SIRP α binding on macrophages	Cancer cells protection from phagocytosis	Increased macrophage phagocytosis by TNF-NF-κB inhibition	[19]
Y239/Y240-Shc-A phosphory-lation	Antitumor STAT-1 activity decrease STAT-3 mediated immune suppression increase	Immunesuppression	Constitutive binding or specific Y239/Y240-Shc-A inhibitors to sensitize to immunotherapies	[25]

G-CSF: granulocyte colony stimulating factor; MDSCs: myeloid derived suppressor cells; INF: interferon; miR: microRNA; EGF: epidermal growth factor; EGFR: epidermal growth factor receptor; FGFR: fibroblast growth factor receptor; PD-L1: programmed death ligand 1; GSK: glycogen synthase kinase; TNF: tumor necrosis factor; NF-κB: nuclear factor kappa B; SEs: super enhancers; LCOR: ligand-dependent nuclear receptor co-repressor; SIRP: signal-regulatory protein; Shc-A: type III chaperone protein ShcA.; STAT: signal transducer and activator of transcription.

**Table 2:** Relationships of the Immune Response (IR) with Tumor Growth and Progression.

IR Factor or Mediator	Mechanism	Result	Perspective	Ref.
CCR7	Membrane CCR7-CCL19/CCL21 interaction; No cytoplasmic CCR7-CCL19/CCL21 interaction	Treg and macrophage attraction to the microenvironment; inversely associated with CD3+ cells in the stroma	Better evaluation of CCR7 role in membrane and cytoplasm	[31]
Annexin-1	FPR2-ERK-NF-κB pathway activation, M2 phenotype macrophages polarization	Angiogenesis, tumor progression, immune suppression	Targeting FPR2-ERK signaling	[41]
NR4A1	NR4A1 hyperexpression T-βRI activation, SMAD 2/3 phosphorylation, intense SMAD signaling	EMT and cell migration, poor prognosis	Targeting TGF-β and NR4A1	[49]

CCR7: C-C motif chemokine receptor 7; CCL: chemokine ligand; FPR: formyl peptide receptor; ERK: extracellular regulated MAP kinase; NF.κB: nuclear factor kappa B; NR4A1: nuclear orphan receptor; TGF: tumor growth factor; T-βRI: TGF-β type I receptor; EMT: epithelial-to-mesenchymal transition.

**Table 3:** The Immune System and Prognosis or Prediction of Response to Therapy.

Immune Genes/s or IR Mediator	Mechanism	Result	Perspective	Ref.
TNBC with BRCA1 dysfunction	“NF-κB on” signal, M1-type macrophages microenvironment, and CD8+ infiltration	Better outcome	Checkpoint inhibitors in addition to conventional FEC CT	[56]
17-gene HTICs signature	IR, proliferation and migration as critical biological pathways	Worse prognosis and benefit from trastuzumab in HER2+ ER- BC	More appropriate adjuvant therapy in HER2+ ER-BC	[57]
LncRNAs	Regulation of the immune system activation by 30 hyper- and 25 hypo- expressed Lnc RNAs	Tumor progression and worse survival	Prognosis and complementation of conventional parameters in specific subtypes	[58]
Immune module SCORE	Activated immune microenvironment	Prediction of response to CT in ER+ and Luminal BCs	Better patient selection and design of combined chemo-immunotherapies	[59]
Immune suppressive plasma cells expressing IGA, IL-10 and PDL-1	Inhibition of oxaliplatin tumor directed CTL activation and ICD	Poor response to oxaliplatin	Inhibition of IGA+ plasmocytes in oxaliplatin treated patients	[65]

TNBC: triple negative breast cancer; BRCA: breast cancer antigen; NF-κB: nuclear factor kappa B; Lnc: long noncoding; BC: breast cancer; FEC: 5-fluorouracil-epirubicin-cyclophosphamide; CT: chemotherapy; HTICS: HER2-TIC-enriched signature; CTL: cytotoxic T lymphocytes; PD-L: programmed death ligand; ICD: immunogenic cell death.
